# 802 Characteristics of Personal Social Network Associated with Social Participation Outcomes After Burn Injury

**DOI:** 10.1093/jbcr/iraf019.333

**Published:** 2025-04-01

**Authors:** Huan Deng, Cailin Abouzeid, Lauren Shepler, Pengsheng Ni, Zachary Wehrwein, Amar Dhand, Mary Slavin, Juan Herrera-Escobar, Lewis Kazis, Colleen Ryan, Jeffrey Schneider

**Affiliations:** Spaulding Rehabilitation Hospital, Harvard Medical School; Spaulding Rehabilitation Hospital, Harvard Medical School; Spaulding Rehabilitation Hospital, Harvard Medical School; Boston University School of Public Health; Brigham and Women’s Hospital, Harvard Medical School; Brigham and Women’s Hospital, Harvard Medical School; Spaulding Rehabilitation Hospital, Harvard Medical School; Brigham and Women’s Hospital, Harvard Medical School; Boston University; Shriners Children’s - Boston and Massachusetts General Hospital; Spaulding Rehabilitation Hospital, Harvard Medical School

## Abstract

**Introduction:**

A personal social network is the set of family, friends and acquaintances surrounding an individual and their interpersonal connections. Personal social networks have shown close associations with an individual’s physical health, mental wellbeing, and social outcomes. People with burn injury commonly experience social participation challenges for years. The association between recovery outcomes and personal social network is unclear. Thus, this study aims to examine the relationship between personal social network characteristics and social participation outcomes after burn injury.

**Methods:**

This is a 6-month prospective study of adult burn survivors living in the community. Participants completed the Personal Network Survey (PERSNET) at baseline, 3-month, and 6-month and the Life Impact Burn Recovery Evaluation (LIBRE) Social Interactions and Social Activities short forms weekly. The PERSNET is an assessment of personal social network and is quantified by two categories of metrics. Network structure (network size, density, and effective network size) describes the arrangement of social connections while network composition (demographic characteristics, relationship type and support type) are the characteristics of network members. The LIBRE is an assessment to measure social participation after burn injury. Multilevel models with random slope and intercept were performed to examine the associations between PERSNET metrics and LIBRE assessment results with adjusting the demographic and burn injury variables.

**Results:**

A total of 23, 21, and 18 participants completed both the PERSNET and LIBRE assessments at baseline, 3-month, and 6-month, respectively. Their average age was 49 years and 70% of them were female with an average 24 years after burn injury and 45% total body surface area burned. The results showed that friends and family were the major relationship types, while camaraderie and emotion were the major support types. Networks with more friends, networks with more persons, and networks with higher effective network size (a network characterized by alters who generally do not know each other) were each associated with the lower scores of LIBRE Social Interactions (worse social participation) (p< 0.05), respectively.

**Conclusions:**

The study findings preliminarily indicate that personal social network structure and relationship type are related to burn survivors’ social participation outcomes.

**Applicability of Research to Practice:**

This study may inform future use of personal social network to identify burn survivors who are prone to social participation challenges.

**Funding for the Study:**

This work was supported by the Stepping Strong Center, Spaulding Research Institute and National Institute on Disability, Independent Living, and Rehabilitation Research.

